# Supplementary material for: COZOID: contact zone identifier for visual analysis of protein-protein interactions
Source: BMC Bioinformatics. 2018 Apr 6;19:125. doi: 10.1186/s12859-018-2113-6 (PMC5889581; doi:10.1186/s12859-018-2113-6)
Supplement: Supplementary file 2 — Software build. Executable binary file of the software tool. (ZIP 118,784 kb) [file 12859_2018_2113_MOESM2_ESM.zip › cozoid/platform/docs/testng-6.5.1-javadoc.zip!/org/testng/SkipException.html]

SkipException (TestNG)


---


|  |  |  |
| --- | --- | --- |
| **Overview** \| **Package** \| **Class** \| **Tree** \| **Deprecated** \| **Index** \| **Help** | |  |
| **PREV CLASS**   **NEXT CLASS** | **FRAMES**    **NO FRAMES**     **All Classes** |  |
| SUMMARY: NESTED \| FIELD \| CONSTR \| METHOD | DETAIL: FIELD \| CONSTR \| METHOD |  |


---


## org.testng Class SkipException

```
java.lang.Object
  java.lang.Throwable
      java.lang.Exception
          java.lang.RuntimeException
              org.testng.SkipException
```

**All Implemented Interfaces:**: java.io.Serializable

**Direct Known Subclasses:**: TimeBombSkipException

---

``` public class SkipException extends java.lang.RuntimeException ```

The root exception for special skip handling. In case a @Test or @Configuration
throws this exception the method will be considered a skip or a failure according to the
return of `isSkip()`.
Users may provide extensions to this mechanism by extending this class.

**Since:**
:   5.6

**Author:**
:   Alex Popescu

**See Also:**: Serialized Form

---

| **Constructor Summary** | |
| --- | --- |
|  | `SkipException(java.lang.String skipMessage)` |
| `protected` | `SkipException(java.lang.String skipMessage, java.lang.Throwable cause)` |


| **Method Summary** | |
| --- | --- |
| `boolean` | `isSkip()`             Flag if the current exception marks a skipped method (true) or a failure (false). |
| `protected  void` | `reduceStackTrace()`             Subclasses may use this method to reduce the printed stack trace. |
| `protected  void` | `restoreStackTrace()`             Restores the original exception stack trace after a previous call to `reduceStackTrace()`. |

**Methods inherited from class java.lang.Throwable**

`fillInStackTrace, getCause, getLocalizedMessage, getMessage, getStackTrace, initCause, printStackTrace, printStackTrace, printStackTrace, setStackTrace, toString`

**Methods inherited from class java.lang.Object**

`clone, equals, finalize, getClass, hashCode, notify, notifyAll, wait, wait, wait`

**Constructor Detail**

### SkipException

```
public SkipException(java.lang.String skipMessage)
```

---


### SkipException

```
protected SkipException(java.lang.String skipMessage,
                        java.lang.Throwable cause)
```


**Method Detail**

### isSkip

```
public boolean isSkip()
```

:   Flag if the current exception marks a skipped method (true)
    or a failure (false). By default Subclasses should override this method
    in order to provide smarter behavior.

    :   **Returns:**: true if the method should be considered a skip, false if the method should be considered failed. If not overwritten it returns true

---


### reduceStackTrace

```
protected void reduceStackTrace()
```

:   Subclasses may use this method to reduce the printed stack trace.
    This method keeps only the last frame.
    **Important**: after calling this method the preserved internally
    and can be restored called `restoreStackTrace()`.

---


### restoreStackTrace

```
protected void restoreStackTrace()
```

:   Restores the original exception stack trace after a
    previous call to `reduceStackTrace()`.


---


|  |  |  |
| --- | --- | --- |
| **Overview** \| **Package** \| **Class** \| **Tree** \| **Deprecated** \| **Index** \| **Help** | |  |
| **PREV CLASS**   **NEXT CLASS** | **FRAMES**    **NO FRAMES**     **All Classes** |  |
| SUMMARY: NESTED \| FIELD \| CONSTR \| METHOD | DETAIL: FIELD \| CONSTR \| METHOD |  |


---
